# A Comprehensive MicroRNA Expression Profile of Liver and Lung Metastases of Colorectal Cancer with Their Corresponding Host Tissue and Its Prognostic Impact on Survival

**DOI:** 10.3390/ijms17101755

**Published:** 2016-10-21

**Authors:** Mathieu Pecqueux, Isabell Liebetrau, Wiebke Werft, Hendrik Dienemann, Thomas Muley, Joachim Pfannschmidt, Benjamin Müssle, Nuh Rahbari, Sebastian Schölch, Markus W. Büchler, Jürgen Weitz, Christoph Reissfelder, Christoph Kahlert

**Affiliations:** 1 Department of General, Visceral and Thoracic Surgery, University of Dresden, Fetscherstraße 74, 01307 Dresden, Germany; Benjamin.Muessle@uniklinikum-dresden.de (B.M.); Nuh.Rahbari@uniklinikum-dresden.de (N.R.); Sebastian.Schoelch@uniklinikum-dresden.de (S.S.); Juergen.Weitz@uniklinikum-dresden.de (J.W.); Christoph.Reissfelder@uniklinikum-dresden.de (C.R.); Christoph.Kahlert@uniklinikum-dresden.de (C.K.); 2 ALB Fils clinic, Eybstr. 16, 73312 Geislingen, Germany; liebetrau.isabell@gmx.de; 3 German Cancer Research Center, Division of Biostatistics, Im Neuenheimer Feld 280, 69120 Heidelberg, Germany; w.werft@hs-mannheim.de; 4 Department of Thoracic Surgery, Thoraxklinik am Universitätsklinikum Heidelberg, Amalienstrasse 5, 69126 Heidelberg, Germany; hendrik.dienemann@med.uni-heidelberg.de (H.D.); Thomas.muley@med.uni-heidelberg.de (T.M.); 5 Department of Thoracic Surgery, Helios Clinic Emil von Behring, Walterhöferstraße 11, 14165 Berlin, Germany; joachim.pfannschmidt@helios-kliniken.de; 6 Department of Surgery at Heidelberg University Hospital, Im Neuenheimer Feld 110, 69120 Heidelberg, Germany; Markus.Buechler@med.uni-heidelberg.de

**Keywords:** miRNA, survival, expression, colorectal, metastasis, metastases

## Abstract

MicroRNAs are small non-coding RNAs with a length of 18–25 nucleotides. They can regulate tumor invasion and metastasis by changing the expression and translation of their target mRNAs. Their expression is substantially altered in colorectal cancer cells as well as in the adjacent tumor-associated stroma. Both of these compartments have a mutual influence on tumor progression. In the development of metastases, cancer cells initially interact with the host tissue. Therefore, compartment-specific expression signatures of these three locations—tumor, associated stroma, and host tissue—can provide new insights into the complex tumor biology of colorectal cancer. Frozen tissue samples of colorectal liver (*n* = 25) and lung metastases (*n* = 24) were laser microdissected to separate tumor cells and the adjacent tumor-associated stroma cells. Additionally, normal lung and liver tissue was collected from the same patients. We performed a microarray analysis in four randomly selected liver metastases and four randomly selected lung metastases, analyzing a total of 939 human miRNAs. miRNAs with a significant change >2-fold between the tumor, tumor stroma, and host tissue were analyzed in all samples using RT-qPCR (11 miRNAs) and correlated with the clinical data. We found a differential expression of several miRNAs between the tumor, the tumor-associated stroma, and the host tissue compartment. When comparing liver and lung metastases, miR-194 showed a 1.5-fold; miR-125, miR-127, and miR-192 showed a 2.5-fold; miR-19 and miR-215 a 3-fold; miR-145, miR-199-3, and miR-429 a 5-fold; miR-21 a 7-fold; and, finally, miR-199-5 a 12.5-fold downregulation in liver metastases compared to lung metastases. Furthermore miR-19, miR-125, miR-127, miR-192, miR-194, miR-199-5, and miR-215 showed a significant upregulation in the normal liver tissue compared to the normal lung tissue. Univariate analysis identified an association of poor survival with the expression of miR-125 (*p* = 0.05), miR-127 (*p* = 0.001), miR-145 (*p* = 0.005), miR-192 (*p* = 0.015), miR-194 (0.003), miR-199-5 (*p* = 0.008), miR-215 (*p* < 0.001), and miR-429 (*p* = 0.03) in the host liver tissue of the liver metastases. Colorectal liver and lung metastases have a unique miRNA expression profile. miRNA expression in the host tissue of colorectal liver metastases seems to be able to influence tumor progression and survival. These findings can be used in the development of tailored therapies.

## 1. Introduction

Progression to metastatic disease is the major cause of cancer-related death in colorectal cancer. At the time of diagnosis 15% of patients have detectable synchronous liver metastases, with 76% of these cases confined to the liver [1]. Eleven percent of patients have synchronous lung metastases, with 61% suffering simultaneously from extrathoracic tumor burden [2]. Several studies have shown the regulatory role of miRNAs in cancer progression and metastasis [3,4]. miRNAs are small, non-coding RNAs that can silence specific target genes by repressing translation [5]. One miRNA can bind up to 1000 different target genes, thereby exerting a strong regulatory function on the cell metabolism. Moreover, depending on the target gene and the cell type, miRNAs can have varying effects on tumor progression by altering the expression of oncogenes and tumor-suppressive genes [6]. Since miRNAs are very resilient against degradation, they are considered a powerful diagnostic tool.

Several studies have investigated the altered expression of miRNAs during the development of colorectal cancer, beginning from normal mucosa via an adenoma to the final stage of cancer [7,8,9]. Recent studies revealed significant differences in the expression profiles of primary colorectal cancers and their corresponding metastases [10]. These studies compared tumor tissue with normal organ tissue. However, tumors and their metastases are complex organ-like structures where tumor cells are embedded into a supporting microenvironment, called a tumor microenvironment. This tumor microenvironment consists of fibroblasts, endothelial cells, pericytes, macrophages, and the extracellular matrix. For the last decade, it has become evident that the tumor microenvironment contributes significantly to the development, progression, and dissemination of colorectal cancer [11]. The tumor microenvironment is essential for the survival of metastases by contributing to its nutritive supply [12]. Furthermore, the implantation of metastases requires a permissive host tissue, the premetastatic niche [13].

In this study, we have focused on the expression profile of miRNAs in colorectal liver and lung metastases. To investigate the impact of the tumor microenvironment, we separated the tumor tissue, the tumor-associated stroma tissue, and the host tissue compartment using laser capture microdissection. Subsequently, the compartment-specific expression profile was evaluated by microarray analysis and RT-qPCR. Furthermore, the results were correlated with clinical data. The separate assessment of the miRNA expression profile in colorectal cancer metastases in the abovementioned compartments can provide useful information in the search of future therapeutic targets.

## 2. Results

### 2.1. Patient Characteristics

The patient characteristics are summarized in Table 1. A total of 50 patients with colorectal metastases were initially included in this study: 25 colorectal liver metastases and 25 colorectal lung metastases. One patient with liver metastases and two patients with lung metastases had to be excluded from the correlation and survival analysis because of missing follow-up data.

The patients with colorectal liver metastases had a mean age of 62 (40–76) years; 16 (67%) were male; 13 (54%) had synchronous metastatic disease; and 11 (46%) had metachronous metastases. The mean progression free survival (PFS) was 23.5 months; the mean overall survival (OS) was 21.7 months.

The patients with colorectal lung metastases had a mean age of 64 (39–79) years; 15 (65%) were male, four (17%) had synchronous metastases; and 19 (83%) had metachronous disease. The mean PFS was 68.6 months and the mean OS was 24.1 months.

### 2.2. miRNA Profiling

The microarray data were evaluated for differentially expressed miRNAs in the tumor, the stroma, and the host tissue compartment. In the liver metastases, a total of 109 miRNAs were differentially expressed between the tumor and the stroma compartment, 20 of which were also differentially expressed in the normal liver tissue. Eleven of these samples showed a >2-fold expression difference between all three compartments (Table S1).

In the lung metastases, 88 miRNAs were differentially expressed between the tumor and the stroma compartment, 12 of which were also differentially expressed in the normal lung tissue. Five of these samples showed a >2-fold expression difference between all three compartments (Table S2). Only three miRNAs, miR-127, miR-192, and miR-215, showed a significant expression difference (>2-fold) between all three compartments in both liver and lung metastases (Tables S1 and S2).

A total of seven miRNAs were selected among the 13 miRNAs with a significant and >2-fold expression difference in liver or lung metastases (Tables S1 and S2). Additionally, several well-known oncogenic/tumor suppressive miRNAs such as miR-19b, miR-21, miR-125b, and miR-429 were included in this study.

The final selection of miRNAs for further analysis consisted of 11 miRNAs: miR-19b, miR-21, miR-125b, miR-127-3p, miR-145, miR-192, miR-194, miR-199a-3p, miR-199a-5p, miR-215, and miR-429. The miRNAs miR-192 and miR-194 are collocated on the miR-192/miR-194-2 cluster on chromosome 11 (11q13.1). miR-194 and miR-215 are situated in the miR-215/miR-194-1 cluster on chromosome 1 (1q41). Therefore we decided to include all three miRNAs in the final analysis. The target sequence for miR-199a-3p also targets miR-199a1, miR199-a2, and miR-199b, whereas the target sequence against miR-199a-5p only binds to miR-199a1 and miR-199a2. Since miR-199a1, miR-199a2, and miR-199b are all located on different chromosomes, we decided to include both miRNAs in the final analysis.

### 2.3. Results of the RT-PCR Analysis: Differential Expression in Tumor, Stroma, and Host Tissue

**Figure 1:**
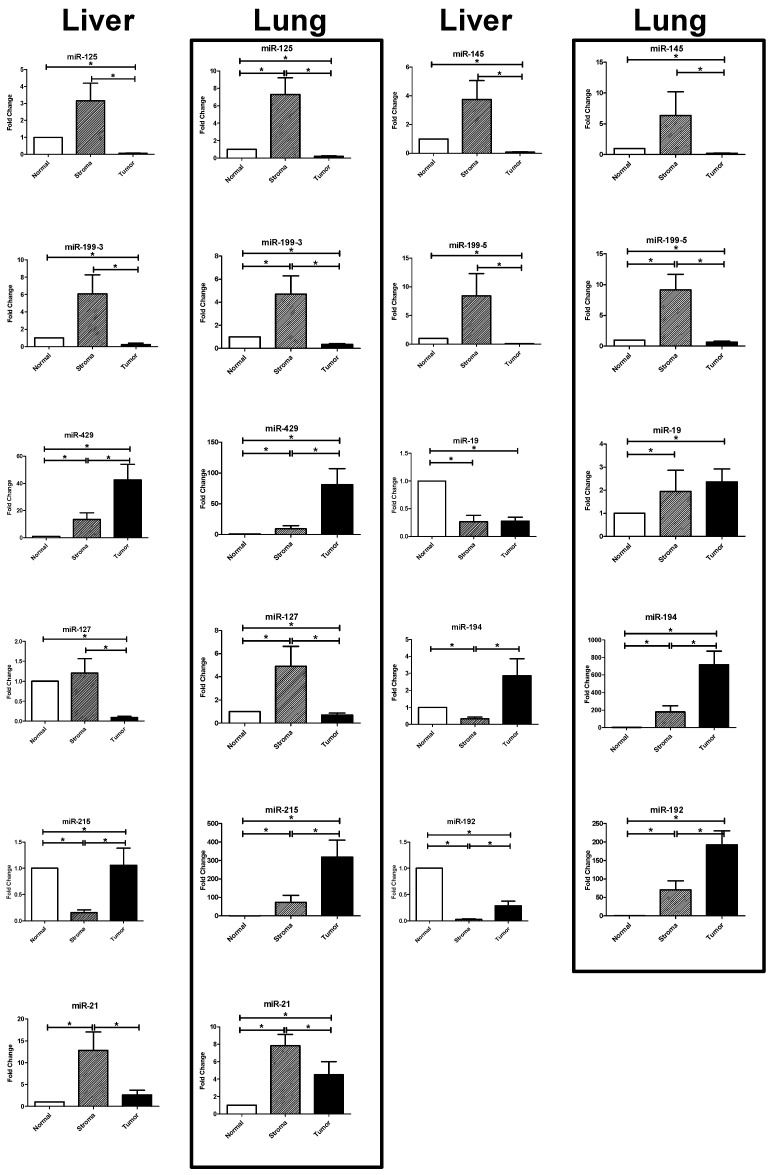
Differential expression between the tumoral, stromal, and host tissue compartment. The graphs show the mean expression difference and the standard error of the difference (SED) as a fold change (ΔΔ*C*_t_-Method) compared to the expression in the normal tissue. *p*-Values were calculated using the *t*-test. Significant expression differences are marked with asterisks (*).

The results of the RT-PCR of the selected miRNAs were analyzed for differential expression in the tumor, the stroma, and the host tissue compartment in 25 patients with colorectal liver metastases and 24 patients with colorectal lung metastases (Figure 1, Table 2). One lung metastasis was excluded because of missing normal tissue.

#### 2.3.1. Upregulated miRNAs in the Tumoral Compartment of Liver and Lung Metastases

We searched for miRNAs that were significantly overexpressed in the tumor compartment. We identified four overexpressed miRNAs in the tumor compartment of liver and lung metastases, respectively (Figure 1, Table 2).

miR-192 showed a 4-fold upregulation in the tumor compartment of the liver metastases (*p* < 0.0001) and a 5-fold upregulation in the tumor compartment of the lung metastases (*p* < 0.0001) compared to the stroma. Compared to the normal tissue, miR-192 showed a significant upregulation in the tumor and the stroma of the lung metastases compared to normal lung tissue (tumor: *p* < 0.0001; stroma *p* = 0.0012). In contrast, miR-192 was significantly downregulated in the tumor and the stroma compartment of the liver metastases compared to normal liver tissue (*p* < 0.0001).

miR-194 showed a 2-fold upregulation in the tumor compartment of the liver metastases compared to the stroma compartment and a 3-fold upregulation compared to the normal liver tissue (*p* < 0.0001). In the lung metastases, miR-194 showed an almost 4-fold upregulation in the tumor compartment compared to the stroma compartment (*p* < 0.0001) and a more than 700-fold upregulation compared to normal lung tissue (*p* < 0.0001).

miR-215 showed a 2.5-fold upregulation in the tumor compartment of the liver metastases compared to the stroma compartment (*p* < 0.0001) and no significant upregulation compared to the normal liver tissue. In the lung metastases miR-215 showed a 10-fold upregulation compared to the stroma and a 300-fold upregulation compared to normal lung tissue (*p* < 0.0001).

miR-429 showed a 4-fold upregulation compared to the stroma tissue and a 46-fold upregulation compared to normal liver tissue (*p* = 0.007 and *p* = 0.0009). In the lung metastases, miR-439 showed a more than 5-fold upregulation in the tumor tissue compared to the stroma tissue and an 80-fold upregulation compared to normal lung tissue (*p* < 0.0001).

#### 2.3.2. Upregulated miRNAs in the Stromal Compartment of Liver and Lung Metastases

Five miRNAs showed a significant upregulation in the stroma compartment (Figure 1, Table 2).

miR-125 showed a 200-fold upregulation in the stroma compartment of the liver metastases compared to the tumor (*p* < 0.0001) but no significant upregulation compared to the normal liver tissue. In the lung metastases, miR-125 showed a 40-fold upregulation in the stroma compartment compared to the tumor compartment (*p* < 0.0001) and a 7-fold upregulation compared to the normal lung tissue (*p* = 0.008).

miR-145 was 200-fold upregulated in the stroma tissue of the liver metastases compared to tumor tissue (*p* < 0.0001) with no significant upregulation compared to the normal liver tissue. In the lung metastases, miR-145 showed a 14-fold upregulation in the stroma compartment compared to the tumor compartment (*p* < 0.0001) with no significant upregulation compared to the normal tissue.

miR-199-3p and miR-199-5p were 500-fold upregulated in the stroma compartment of the liver metastases compared to the tumor compartment (*p* < 0.0001), respectively, but did not show a significant upregulation compared to the normal liver tissue. In the lung metastases, miR-199-3p showed a 14-fold and miR-199-5p showed a 25-fold upregulation in the stroma compartment compared to the tumor compartment (*p* < 0.0001) and a 5-fold (miR199-3p; *p* = 0.03) and 9-fold (miR-199-5p; *p* = 0.0004) upregulation compared to the normal lung tissue.

miR-127 was 60-fold upregulated in the stroma tissue compared to the tumor tissue of the liver metastases (*p* < 0.0001) but showed no significant upregulation compared to the normal liver tissue. miR-127 showed a 10-fold upregulation in the stroma compartment of the lung metastases compared to the tumor compartment (*p* < 0.0001) and a 5-fold upregulation compared to the normal lung tissue (*p* = 0.01).

miR-21 was 30-fold upregulated in the stroma tissue compared to the tumor tissue of the liver metastases (*p* < 0.0001) and was 14-fold upregulated compared to the normal liver tissue (*p* = 0.0006). In the lung metastases, miR-21 showed a 4-fold upregulation in the stroma compared to the tumor tissue (*p* = 0.005) and a 12-fold upregulation compared to the normal lung tissue (*p* = 0.0001).

#### 2.3.3. Differential Expression between Liver and Lung Metastases

**Figure 2:**
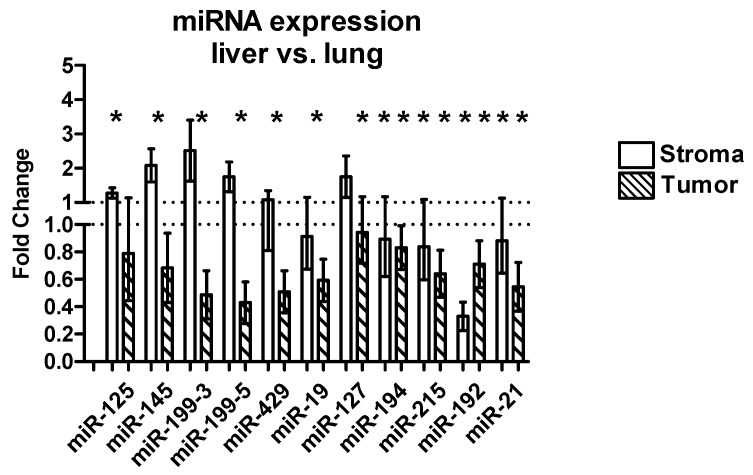
Differential expression between liver and lung metastases. The graph shows the mean fold change and the standard error of the mean SEM (ΔΔ*C*_t_-Method) of the stromal tissue in the liver metastases compared to the stromal tissue in the lung metastases (empty bars) and the tumor tissue in the liver metastases compared to the tumor tissue in the lung metastases (hashed bars). *p*-values were calculated using the *t*-test. Significant expression differences are marked with an asterisk (*).

We compared the expression of miRNAs between liver and lung metastases, separately for tumor and stroma tissue (Figure 2, Table 3). The Δ cycle-threshold-values (Δ*C*_t_-values) in the liver metastases were compared to the Δ*C*_t_-values in the lung metastases (*t*-test) and the fold change was calculated using the comparative Δ*C*_t_ method (ΔΔ*C*_t_-Method).

We found a significant downregulation of all miRNAs in the tumor compartment of the liver metastases compared to the lung metastases. miR-194 showed a 1.5-fold; miR-125, miR-127, and miR-192 showed a 2.5-fold; miR-19 and miR-215 a 3-fold; miR-145, miR-199-3, and miR-429 a 5-fold; miR-21 a 7-fold; and miR-199-5 a 12.5-fold downregulation in the liver metastases compared to the lung metastases.

In the tumor stroma only miR-19, miR-215, and miR-21 showed a significant downregulation in the liver metastases compared to the lung metastases, but none of the miRNAs was downregulated more than by 2-fold.

In the host tissue of the liver and the lung metastases, several miRNAs showed a significant upregulation in the liver metastases compared to the lung metastases.

**Figure 3:**
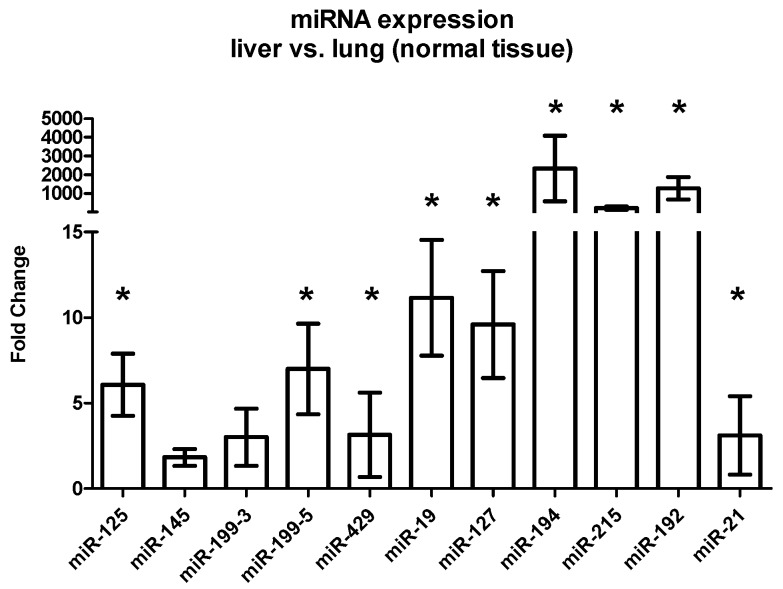
Differential expression between the host tissue of colorectal liver and lung metastases. The graph shows the mean fold change with the standard error of the mean SEM (ΔΔ*C*_t_-Method) of the host tissue in the liver metastases compared to the expression of the host tissue in the lung metastases. *p*-values were calculated using the *t*-test. Significant expression differences are marked with an asterisk (*).

miR-125 and miR-199-5 showed a 2-fold; miR-19 and miR-127 showed a 4-fold; miR-215 showed a 100-fold; miR-194 showed a 150-fold; and miR-192 showed a 300-fold upregulation in the normal liver tissue compared to the normal lung tissue. The expressions of miR-145, miR-199-3, miR-429, and miR-21 showed similar results in liver and lung tissue (Figure 3, Table 3).

### 2.4. Survival Data

The RT-PCR data was dichotomized into samples with low expression and high expression according to the median. Survival analyses were performed using Kaplan Meier curves and *p*-values were calculated using the Log-rank test.

#### Univariate Survival Analysis

The univariate analyses showed no significant association between miRNA levels in the tumor compartment of the liver metastases and survival. In the stroma compartment of the liver metastases, we found a significant association between the down regulation of miR-199-3 and poor survival (*p* = 0.05).

In the host tissue of the liver metastases, we identified several miRNAs with significant correlations between expression and survival: downregulation of miR-125 (*p* = 0.05), miR-127 (*p* = 0.001), miR-145 (*p* = 0.005), miR-192 (*p* = 0.015), miR-194 (0.003), miR-199-5 (*p* = 0.008), miR-215 (*p* < 0.001), and miR-429 (*p* = 0.03) was associated significantly with poor survival (Table 4).

In the lung metastases, none of the miRNAs was significantly associated with survival.

**Figure 4:**
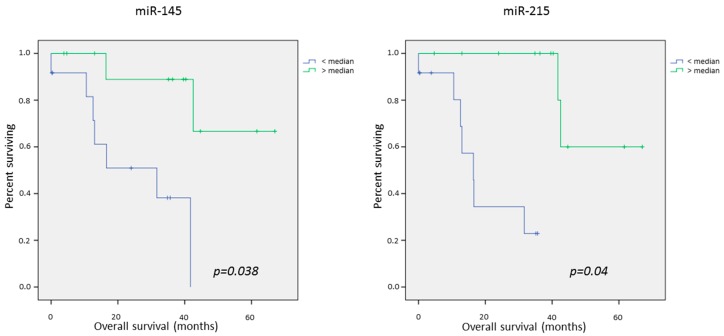
Kaplan–Meier curves of significant factors (miR-145 and miR-215 in the host tissue of the liver metastases) for overall survival in lung and liver Metastases.

When combining the results of the expression of miRNAs in liver and lung metastases, only the expression of miR-145 and miR-215 in the host tissue was significantly associated with overall survival (*p* = 0.038 and *p* = 0.04, Figure 4).

## 3. Discussion

This is the first study comparing separately the expression profile of miRNAs of colorectal liver and lung metastases in the tumor, the stroma, and the host tissue compartment.

Tumors are complex organ-like structures. The surrounding microenvironment that supports tumor growth and metastasis takes part in creating a complex mosaic of intratumoral variation with selective tensions such as hypoxia, acidity, or cytokine expression [14]. The separate analysis of the tumor, the stroma, and the host tissue compartments allows conclusions about the interactions between these compartments and permits a more accurate evaluation of the tumor profile.

This study identified a differential expression profile of miRNAs between the tumor and stroma compartment within the metastases. We found a significant upregulation of miR-429 in the tumor compartment of both liver and lung metastases, indicating an oncogenic role of this micro-RNA. These results are in good accordance with existing findings about the oncogenic role of miR-429 in colorectal cancers [3,15]. Furthermore, we found a significant upregulation of miR-19, miR-192, miR-194, and miR-215 in the tumor compartment of the lung metastases and a significant downregulation of the same miRNAs in the liver metastases. These data may reflect the ambivalent role of miRNAs in tumor progression. In fact, oncogenic as well as tumor suppressive properties have been assigned to some of these miRNAs [16,17,18,19]. miR-192 and miR-215 have been shown to be upregulated by p53, a tumor suppressor. Both miRNAs can induce cell-cycle arrest and apoptosis [16]. The upregulation in the tumor compartment of the lung metastases suggests that these miRNAs could have additional oncogenic functions. There are multiple ways to regulate miRNA function after biosynthesis [20]. The function of these miRNAs seems to be dependent from environmental factors. Thus, it can be conjectured that the oncogenic/tumor suppressive function could be influenced by the host tissue. In this light, those data could explain the conflicting results about organ-dependent differential expression of miRNAs in our study.

Next, this report identified a differential expression of miRNAs between liver and lung metastases. Several miRNAs were significantly downregulated in the tumor compartment of the liver metastases compared to the lung metastases, whereas the stromal compartments showed similar expressions. A comparison of the normal liver and lung tissue revealed a significant upregulation of several miRNAs in the liver tissue. Noteworthy, miR-215, miR-194, and miR-192 showed a more than 100-fold upregulation in the normal liver tissue compared to the normal lung tissue. These results are congruent with earlier data by our group [21] that indicate that the protein expression profile of colorectal metastases is dependent on the host tissue and might be strongly influenced by the surrounding host tissue.

Finally we performed survival analyses to elucidate the impact of the expression of miRNAs on survival. Intriguingly, we found that the expression of miR-199-3p in the stromal compartment of liver metastases was significantly associated with an improved survival (*p* = 0.05; Table 4). miR-199a can downregulate mTOR (mechanistic Target of Rapamycin), c-MET (protein encoded by the MET gene), and its downstream effector ERK2, thus inhibiting cell proliferation, motility, and the invasive capabilities of tumor cells [22,23]. Both miR-125 and miR-199a were shown to inhibit angiogenesis through decreased expression of HIF‑1a (Hypoxia-inducible factor 1-alpha) and VEGF (Vascular Endothelial Growth Factor) in ovarian cancer [24]. It remains speculative but those data might explain why an increased expression of miR-199-3p in the stromal compartment is associated with a better clinical outcome.

While none of the miRNA expression in the tumor and stroma compartment of lung metastases was associated with the prognosis, we observed multiple significant correlations between the survival and the expression of miRNAs in the host tissue of the liver metastases.

Downregulation of miR-125 (*p* = 0.05), miR-127 (*p* = 0.001), miR-145 (*p* = 0.005), miR-192 (*p* = 0.015), miR-194 (*p* = 0.003), miR-199-5 (*p* = 0.008), miR-215 (*p* < 0.001), and miR-429 (*p* = 0.03) in the normal liver tissue was significantly associated with poor survival, suggesting oncosuppressive effects of these miRNAs. miR-125b has been shown to have oncosuppressive effects in hepatocellular cancer [25] as well as in cutaneous squamous cell carcinomas [26]. miR-145 is a target of p53 and has been shown to repress c-Myc [27]. Furthermore, miR-145 can inhibit angiogenesis through post-transcriptional regulation of N-RAS and VEGF-A [28]. Our findings show consistent results with a downregulation of miR-125 and miR-145 in the cancer compartment.

miR-127 acts as a tumor suppressor by downregulating BCL6. BCL6 constrains the plasticity of T-helper cells through attenuating the differentiation of regulatory T-cells (Treg) into follicular T-helper cells [29]. miR-127 has been shown to exert tumor suppressive functions in gastric cancers by interacting directly with the mRNA-encoding oncogenic factors KRAS and MAPK4 [30]. miR-192, -194, and -215 are induced by p53, a well-known tumor suppressor, and influence cell proliferation through the induction of cell cycle arrest. miR-192 and -215 have been found to be downregulated in primary colorectal cancers [16]. Furthermore all three miRNAs can also increase p53 expression through an autoregulatory loop, which could explain their antitumoral effect [31]. miR-192, -194 and -215 are located in the miR-215/miR-194-1 cluster on chromosome 1 (1q41) and the miR-192/miR-194-2 cluster on chromosome 11 (11q13.1). This likely explains a similar expression pattern in our study.

Our results show a downregulation of miR-192, miR-194, and miR-215 in the tumor and the tumor-associated stromal compartment of the liver metastases. Yet, we could also observe an upregulation in the tumor and stroma compartment of the lung metastases. These contradictory results could be explained by the 1000-fold and 300-fold downregulation of miR-192, miR-194, and miR-215 in the host tissue of the lung compared to the liver, thus resulting in a relative upregulation in the tumor and stroma compartment.

miR-194 is expressed in the liver parenchyma and can prevent metastasis [32]. Moreover, miR-194 has been shown to suppress metastasis in non-small lung cancer through suppression of TGFβ activity via suppression of bone morphogenetic protein 1 (BMP1) as well as suppression of cyclin-dependent kinase inhibitor 1B (CDKN1B), which controls the cell cycle [33]. miR-429 is an oncogene, which was shown to inhibit apoptosis in colorectal cancer via SOX2 and can induce epithelial to mesenchymal transition [3,34]. Our data are in line with these features of miR-429 by showing a significant overexpression of miR-429 in the tumor compartment compared to normal tissue and the stroma compartment in the liver and lung metastases. Yet, we could also show a significant association of miR-429 expression in the host tissue of the liver metastases with better survival, suggesting that miR-429 could also have a different, tumor-protective function in the host tissue.

One important question remains: why could this effect only be observed in the liver metastases? When comparing miRNA expression levels in the liver and lung metastases, we could show a significant upregulation in the liver metastases or rather a downregulation in the lung metastases (Figure 2). Especially miR-192, miR-194, and miR-215 were downregulated up to 350 times in the lung metastases compared to the liver metastases. These findings might explain why the further downregulation of these factors does not seem to play a role in the host tissue of lung metastases.

These findings could be interesting when selecting treatments for patients that include targeted therapies.

Many studies have explored the expression profile of the primary colorectal tumor compared to the tissue of origin to assess the expression changes in tumors. Yet, cancer death almost always occurs due to metastatic disease. In the current study, we show that the expression profile of the liver tissue is associated with survival in patients with colorectal liver metastases. Furthermore, the expression profile of different host tissues is significantly different from one another. Taken together, these results suggest an important role of the metastatic host tissue in the progression of metastases. These factors should be taken into account when searching for new targeted therapies, but also when selecting targeted therapies for individual patients.

It is yet unclear how much of these expression changes are innate to the host tissue, and how much is influenced by the tumor. Previous studies could show that in metastatic colorectal cancer, the primary tumor cells can influence the expression of angiogenic factors such as VEGF in the normal liver tissue, thus influencing metastatic growth [35].

Furthermore, recent publications suggest a connection between miRNA expression and resistance to chemotherapy [4,36]. Chemotherapy is the main treatment for metastatic disease, including metastasized colorectal cancers. Many curatively resected cancers need neoadjuvant or adjuvant therapy to ensure treatment success. The tumor-associated stroma has been shown to play an important role in chemoresistance [37]. The significant expression differences in the different host tissues of metastases imply that host tissues play a role in the reaction to chemotherapy and thus influence chemoresistance.

These findings confirm the complexity of cancer, which not only consists of the tumor cells, but also includes the interactions of the cancer cells with the surrounding tissue cells including the tumor-associated stroma, and the host tissue in metastases. The impact of the host tissue on metastatic development, growth, and chemoresistance, as well as the influence of the primary tumor on the expression of oncogenic factors, needs to be further investigated to better understand the relevance of differential host tissue expression on metastatic behavior.

In conclusion, we provide evidence that the miRNA expression in the host tissue of liver metastases plays an influential role on tumor progression and influences survival.

## 4. Materials and Methods

### 4.1. Tumor Samples and Clinical Data

Kryo-frozen tissue samples of colorectal liver metastases were obtained from 25 patients undergoing tumor resection between the years 2004 and 2009 at the Department of General, Visceral, and Transplantation Surgery, University of Heidelberg, Heidelberg, Germany. Cryo-frozen tissue samples of colorectal lung metastases were retrieved from 25 patients undergoing tumor resection between the years 2003 and 2008 at the Department of Thoracic Surgery, University of Heidelberg. Tissue collection was approved by the Ethics Committee of the University of Heidelberg and a written informed consent was obtained from all patients prior to tissue collection. Clinical information including age, gender, TNM classification of the primary tumor, time of occurrence (synchronous vs. metachronous), and overall survival (time from diagnosis to death or last follow-up) were collected for all patients (Table 1).

### 4.2. Clinical Specimens

Tissue collection was performed as described previously [38]. Briefly, samples were snap-frozen in liquid nitrogen immediately after tumor resection and stored at −80 °C until further processing.

### 4.3. Tissue Preparation and Laser Microdissection

**Figure 5:**
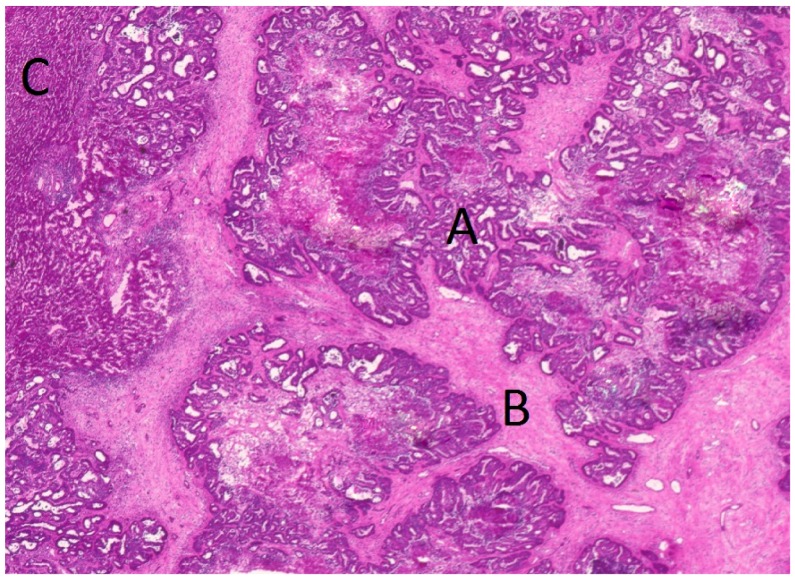
Hematoxylin and eosin (H-E) staining of a liver metastasis: A = tumor tissue, B = stroma tissue, C = liver host tissue. Scanned by Nikon Coolscan 5000 ED at 4000 dpi (Nikon, Tokyo, Japan).

Prior to microdissection, 10-µm sections were cut from the frozen tissue samples using a cryostat (Leica, Wetzlar, Germany) and stained with hematoxylin and eosin by standard methods. These sections were used to ensure the presence of tumor tissue in the samples and to define dissection margins (Figure 5).

For laser-microdissection, 20-µm sections of tissue samples were mounted on Zeiss membrane slides (Carl Zeiss microimaging, Jena, Germany) and stained with cresyl violet using an laser capture microdissection Staining Kit (Ambion^®^/Applied Biosystems, Darmstadt, Germany). Subsequently, laser-microdissection was conducted using a PALM^®^ MicroBeam Laser System (PALM^®^ Microlaser Technologies AG, Bernried, Germany) to separate tumor cells from stromal cells. Tissue from the respective host organs (liver and lung parenchyma) was collected at least 3 cm away from the tumor bulk. Microdissected tissue was transferred to adhesive caps (Carl Zeiss, Jena, Germany), lysed in QIAzol^®^ (Qiagen, Hilden, Germany), and stored at −80 °C until final evaluation.

### 4.4. RNA Extraction

Total RNA, including miRNA, was extracted (miRNeasy, Qiagen) and the RNA integrity was evaluated (Agilent^®^ RNA 6000 Pico Kit, Agilent Technologies, Santa Clara, CA, USA) according to the manufacturer’s instructions. Samples were included if the RNA integrity number was above 7, as recommended [39].

### 4.5. Microarray Analysis

Microarray analysis was performed for four randomly selected samples of colorectal liver and four samples of colorectal lung metastases. Each sample was microdissected into tumor, stroma, and host tissue, as mentioned before. The total RNA concentration in the dissected samples had to be increased to 100 ng/µL using a vacuum concentrator. The concentration of all samples was quantified by NanoDrop 1000 (Nanodrop, Wilmington, DE, USA). RNA integrity was again measured after concentration to ensure persistent quality.

The microarray (Agilent human miRNA Microarray Release 12.0) analyzed a total of 939 human miRNAs (866 human miRNAs and 89 human viral miRNAs). The microarray was processed according to the manufacturer’s instructions.

### 4.6. Real-Time Quantitative PCR (qRT-PCR)

Total RNA, including miRNA, was converted to cDNA (miScript RT Kit, Qiagen, see Table S3) and adjusted at a concentration of 5 ng/µL. Real-time polymerase chain reaction (RT-PCR) was performed using commercially available primers (Qiagen, Hilden, Germany) for the identified miRNAs as well as some previously described miRNAs (Light Cycler™; Roche Diagnostics GmbH).

The cycle threshold (*C*_t_) describes the number of amplification cycles necessary for the fluorescence signal to significantly exceed the background fluorescence. ∆*C*_t_s were calculated using the reference gene RNU6B.

### 4.7. Statistical Analysis

All statistical analyses were performed using the SPSS software version 20 (IBM, New York, NY, USA). Expression differences between the tumoral, the stromal and the host tissue compartment were calculated using the *t*-test [40]. *p*-values < 0.05 were regarded as significant. Fold changes between the different compartments were calculated using the comparative Δ*C*_t_ method (ΔΔ*C*_t_-method).

The influence of miRNAs on overall survival was assessed by univariate analysis using Kaplan–Meier curves and the log-rank test.

## Figures and Tables

**Table 1:** Descriptive data of included samples.

Characteristic		Liver Metastases (*n* = 24)	Lung Metastases (*n* = 23)
Age (years)	min–max	40–76	36–79
	mean ± SD	62 ± 10.32	64 ± 10.78
Sex	male	16	15
	female	8	8
Metastases	synchron.	13	4
	metachron.	11	19
PFS (months)	mean ± SD	23.51 ± 26.18	68.60 ± 41.26
	median	13.59	54.58
Events OS	total	9	16
	%	37.5	69.6
OS (months)	mean ± SD	21.68 ± 15.26	24.11 ± 15.89
	Median	16.51	22.75

PFS: progression free survival; OS: overall survival; SD: standard deviation.

**Table 2:** Differential expression between the tumor (T), the stroma (S), and the normal tissue (N) compartment (fold change, *p*-values were calculated using a Wilcoxon signed-rank test).

Differential Expression in Liver and Lung Metastases							
miRNA	Tissue	S vs. N	*p*-Value	T vs. N	*p*-Value	S vs. T	*p*-Value
miR-125b	liver	3.417	0.438	0.070	<0.0001	211.500	<0.0001
	lung	7.305	0.0078	0.206	<0.0001	43.990	<0.0001
miR-145	liver	4.048	0.533	0.095	<0.0001	223.100	<0.0001
	lung	1.848	0.3465	0.203	<0.0001	14.560	<0.0001
miR-199a-3p	liver	6.593	0.07	0.249	<0.0001	624.400	<0.0001
	lung	4.700	0.0296	0.345	<0.0001	14.480	<0.0001
miR-199a-5p	liver	9.137	0.0803	0.071	<0.0001	510.900	<0.0001
	lung	9.143	0.0004	0.649	0.0016	25.560	<0.0001
miR-429	liver	14.610	0.0065	46.150	0.0009	0.25	0.0066
	lung	9.418	0.0163	80.890	<0.0001	0.178	<0.0001
miR-127-3p	liver	1.310	0.0658	0.101	<0.0001	61.180	<0.0001
	lung	4.907	0.0149	0.702	0.0023	10.950	<0.0001
miR-19b	liver	0.278	<0.0001	0.291	<0.0001	2.569	0.2699
	lung	1.944	0.0006	2.326	0.0007	1.475	0.0383
miR-194	liver	0.349	<0.0001	3.103	0.7265	0.466	<0.0001
	lung	168.500	<0.0001	714.900	<0.0001	0.272	<0.0001
miR-215b	liver	0.167	<0.0001	1.147	0.0372	0.392	<0.0001
	lung	58.310	0.0028	319.100	<0.0001	0.095	<0.0001
miR-192	liver	0.030	<0.0001	0.306	<0.0001	0,255	<0.0001
	lung	33.480	0.0012	192.100	<0.0001	0,198	<0.0001
miR-21	liver	13.900	0.0006	2.872	0.2296	33.680	<0.0001
	lung	11.880	0.0001	5.653	0.0025	4.557	0.0045

**Table 3:** Differential expression of miRNAs in the tumor, the stroma, and the host tissue of liver and lung metastases (mean and standard error of the mean (SEM)). Positive values indicate upregulation; negative values indicate downregulation. *p*-values were calculated using the *t*-test. FC: fold change.

miRNA	Tissue	FC Liver vs. Lung (Mean)	SEM	*p*-Value
miR-125	Stroma	1.00	0.14	0.80
	Tumor	−2.5	1.01	0.003
	Host tissue	1.86	1.82	0.013
miR-145	Stroma	2.33	0.79	0.54
	Tumor	−5.5	0.22	0.0002
	Host tissue	−1.2	0.49	0.51
miR-199-3	Stroma	1.32	1.04	0.72
	Tumor	−5.00	0.16	<0.0001
	Host tissue	1.06	1.68	0.73
miR-199-5	Stroma	1.32	0.47	0.85
	Tumor	−12.5	0.14	<0.0001
	Host tissue	2.85	2.64	0.008
miR-429	Stroma	−1.56	0.30	0.26
	Tumor	−5.56	0.14	0.0002
	Host tissue	−2.22	2.47	0.017
miR-19	Stroma	−1.56	0.30	0.04
	Tumor	−3.57	0.19	0.0001
	Host tissue	3.70	3.38	0.0001
miR-127	Stroma	1.05	0.56	0.91
	Tumor	−2.38	0.25	0.02
	Host tissue	4.18	3.13	<0.0001
miR-194	Stroma	−2.63	0.46	0.03
	Tumor	−1.59	0.17	0.03
	Host tissue	156	1742	<0.0001
miR-215	Stroma	−1.72	0.37	0.01
	Tumor	−3.33	0.21	0.0004
	Host tissue	108	83.25	<0.0001
miR-192	Stroma	−3.33	0.18	<0.0001
	Tumor	−2.71	0.18	0.001
	Host tissue	347	598	<0.0001
miR-21	Stroma	−1.92	0.21	0.04
	Tumor	−7.14	0.17	<0.0001
	Host tissue	−1.89	2.30	0.18

**Table 4:** Univariate analysis of overall survival (log rank). Significant *p*-values (<0.05) are written in bold (ds = stromal tissue, dt = tumor tissue, dn = normal tissue).

miRNA	Liver			Lung			Liver and Lung		
	<median	>median	*p*	<median	>median	*p*	<median	>median	*p*
miR-125ds	34.9	46.2	0.25	37.9	31.4	0.57	36.6	39.3	0.70
miR-125dt	36.0	38.8	0.28	30.9	36.9	0.82	33.8	43.7	0.40
miR-125dn	27.2	56.9	**0.05**	32.8	37.0	0.71	32.5	45.3	0.12
miR-145ds	41.3	43.1	0.84	36.6	34.1	0.67	37.9	39.3	0.98
miR-145dt	46.0	31.2	0.46	36.3	33.4	0.74	41.0	34.6	0.36
miR-145dn	25.3	55.9	**0.005**	33.5	34.7	0.75	31.4	45.6	**0.038**
miR-199-3ds	27.9	54.7	**0.05**	35.6	35.3	0.76	33.0	45.3	0.23
miR-199-3dt	43.2	32.8	0.98	32.2	38.6	0.69	37.0	41.4	0.79
miR-199-3dn	28.9	51.1	0.08	35.1	34.5	0.76	33.0	44.2	0.42
miR-199-5ds	41.3	43.1	0.84	36.0	33.8	0.67	38.5	38.8	0.85
miR-199-5dt	41.5	33.6	0.81	35.6	33.5	0.62	38.4	39.0	0.87
miR-199-5dn	25.9	55.9	**0.008**	41.2	30.4	0.39	35.3	43.0	0.28
miR-429ds	40.9	41.7	0.83	37.4	32.4	0.65	39.6	36.7	0.88
miR-429dt	45.2	31.5	0.56	35.9	35.0	0.98	40.6	36.7	0.73
miR-429dn	27.4	55.4	**0.03**	30.6	41.2	0.43	31.1	49.3	0.06
miR-19ds	42.6	42.8	0.97	32.5	36.1	0.69	37.1	39.5	0.77
miR-19dt	45.0	32.0	0.59	39.4	31.1	0.54	43.8	32.4	0.33
miR-19dn	28.9	51.1	0.12	34.1	36.7	0.75	34.2	43.6	0.21
miR-127ds	35.2	46.7	0.48	32.0	38.1	0.59	33.1	44.3	0.32
miR-127dt	44.5	33.1	0.68	38.5	27.4	0.30	42.3	30.6	0.34
miR-127dn	22.3	57.1	**0.001**	38.8	31.7	0.62	33.7	44.1	0.12
miR-194ds	42.1	41.0	0.97	35.0	34.2	0.96	39.0	37.5	0.88
miR-194dt	39.6	36.1	0.52	28.2	34.3	0.18	33.1	46.8	0.16
miR-194dn	24.5	56.1	**0.003**	40.6	27.0	0.42	34.7	42.4	0.19
miR-215ds	41.8	38.3	0.68	37.9	30.9	0.52	41.0	35.3	0.39
miR-215dt	47.6	37.7	0.48	36.7	31.9	0.56	40.8	36.4	0.44
miR-215dn	19.7	57.1	**0.000**	34.3	34.3	0.85	23.4	47.8	**0.04**
miR-192ds	43.5	39.5	0.72	35.1	34.3	0.99	40.5	37.8	0.76
miR-192dt	46.0	35.1	0.88	29.9	39.9	0.44	36.0	41.3	0.69
miR-192dn	26.0	55.9	**0.015**	44.4	24.9	0.13	37.8	40.3	0.58
miR-21ds	44.1	33.1	0.56	38.4	29.0	0.45	42.2	31.8	0.42
miR-21dt	52.0	31.8	0.29	28.4	41.3	0.33	36.5	39.7	0.96
miR-21dn	31.8	50.2	0.20	40.7	27.0	0.35	38.9	39.4	0.83

## Supplementary Materials

Supplementary materials can be found at www.mdpi.com/1422-0067/17/10/1755/s1.
