# Supplementary material for: A Comprehensive MicroRNA Expression Profile of Liver and Lung Metastases of Colorectal Cancer with Their Corresponding Host Tissue and Its Prognostic Impact on Survival
Source: Int J Mol Sci. 2016 Oct 21;17(10):1755. doi: 10.3390/ijms17101755 (PMC5085780; doi:10.3390/ijms17101755)
Supplement: Supplementary file 1 [file ijms-17-01755-s001.pdf]

Supplementary Materials: A Comprehensive Microrna Expression Profile of Liver- and Lung-Metastases of Colorectal Cancer with Their Corresponding Host Tissue and Its Prognostic Impact on Survival

Mathieu Pecqueux, Isabell Liebetrau, Wiebke Werft, Hendrik Dienemann, Thomas Muley, Joachim Pfannschmidt, Benjamin Müssle, Nuh Rahbari, Sebastian Schölch, Markus W. Buechler, Juergen Weitz, Christoph Reissfelder and Christoph Kahlert

Table S1. Differentially regulated miRNAs between tumoral, stromal and normal tissue calculated using the $\Delta\Delta C_t$-method (≥2-fold change; $p < 0.05$) in the liver-metastases. (Svs.N = stroma compared to host tissue; Svs.T = stroma vs. tumor tissue).

**Liver-Metastases**				
**miRNA**	**Fold Change Svs.N**	***p*-Value**	**Fold Change Svs.T**	***p*-Value**
miR-127-3p	−1.912	0.036	1.917	0.008
miR-192	3.515	0.042	−4.443	0.014
miR-215	3.595	0.043	−4.748	0.014
miR-200c	−2.579	0.036	−4.647	0.017
miR-143	−2.173	0.036	2.206	0.013
miR-194	3.957	0.045	−4.894	0.026
miR-199b-5p	−3.547	0.012	2.899	0.007
miR-145	−2.579	0.045	3.031	0.005
miR-199a-3p	−2.634	0.012	3.459	0.014
miR-214	−3.083	0.010	3.710	0.005
miR-199a-5p	−2.424	0.009	3.741	0.004

Table S2. Differentially regulated miRNAs between tumoral, stromal and normal tissue using the $\Delta\Delta C_t$-method (≥2-fold change; $p < 0.05$) in the lung-metastases. (Svs.N = stroma compared to host tissue; Svs.T = stroma vs. tumor tissue).

**Lung-Metastases**				
**miRNA**	**Fold Change Svs.N**	***p*-Value**	**Fold Change Svs.T**	***p*-Value**
miR-127-3p	−1.973	0.041	2.062	0.023
miR-192	−2.571	0.028	−3.449	0.022
miR-215	−2.312	0.034	−3.455	0.023
miR-141	2.156	0.042	−3.835	0.006
miR-375	2.080	0.009	−2.844	0.002

Table S3. Primer List.

Primer	Sequence 5′-3′	Sanger Accession	Manufacturer
Hs_RNU6B_2			QIAGEN GmbH, Hilden, Germany
hsa-miR-19b	UGUGCAAAUCCAUGCAAAACUGA	MIMAT0000074	QIAGEN GmbH, Hilden, Germany
hsa-miR-21	UAGCUUAUCAGACUGAUGUUGA	MIMAT0000076	QIAGEN GmbH, Hilden, Germany
hsa-miR-127-3p	UCGGAUCCGUCUGAGCUUGGCU	MIMAT0000446	QIAGEN GmbH, Hilden, Germany
hsa-miR-125b	UCCCUGAGACCCUAACUUGUGA	MIMAT0000423	QIAGEN GmbH, Hilden, Germany
hsa-miR-145	GUCCAGUUUUCCCAGGAAUCCCU	MIMAT0000437	QIAGEN GmbH, Hilden, Germany
hsa-miR-192	CUGACCUAUGAAUUGACAGCC	MIMAT0000222	QIAGEN GmbH, Hilden, Germany
hsa-miR-199a1/a2-5p	CCCAGUGUUCAGACUACCUGUUC	MIMAT0000231	QIAGEN GmbH, Hilden, Germany
hsa-miR-194	UGUAACAGCAACUCCAUGUGGA	MIMAT0000460	QIAGEN GmbH, Hilden, Germany
hsa-miR-199a1/a2/b-3p	ACAGUAGUCUGCACAUUGGUUA	MIMAT0000232	QIAGEN GmbH, Hilden, Germany
hsa-miR-215	AUGACCUAUGAAUUGACAGAC	MIMAT0000272	QIAGEN GmbH, Hilden, Germany
hsa-miR-429	UAAUACUGUCUGGUAAAACCGU	MIMAT0001536	QIAGEN GmbH, Hilden, Germany
